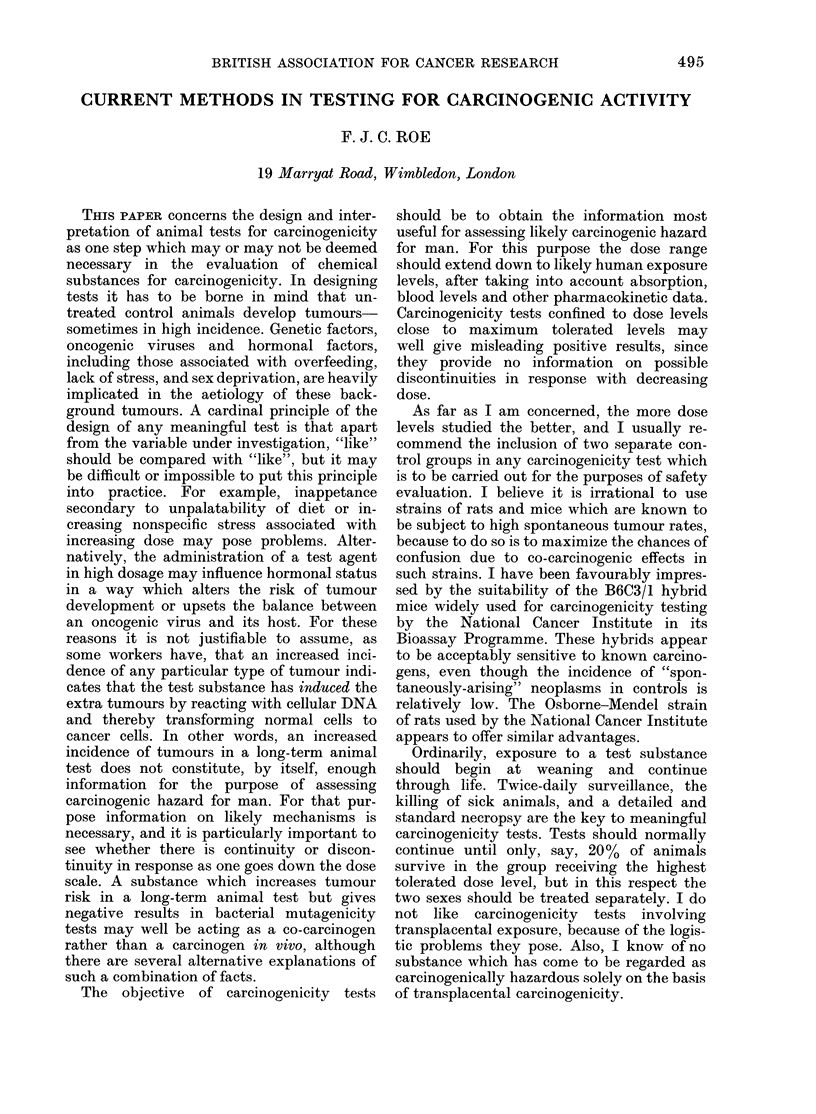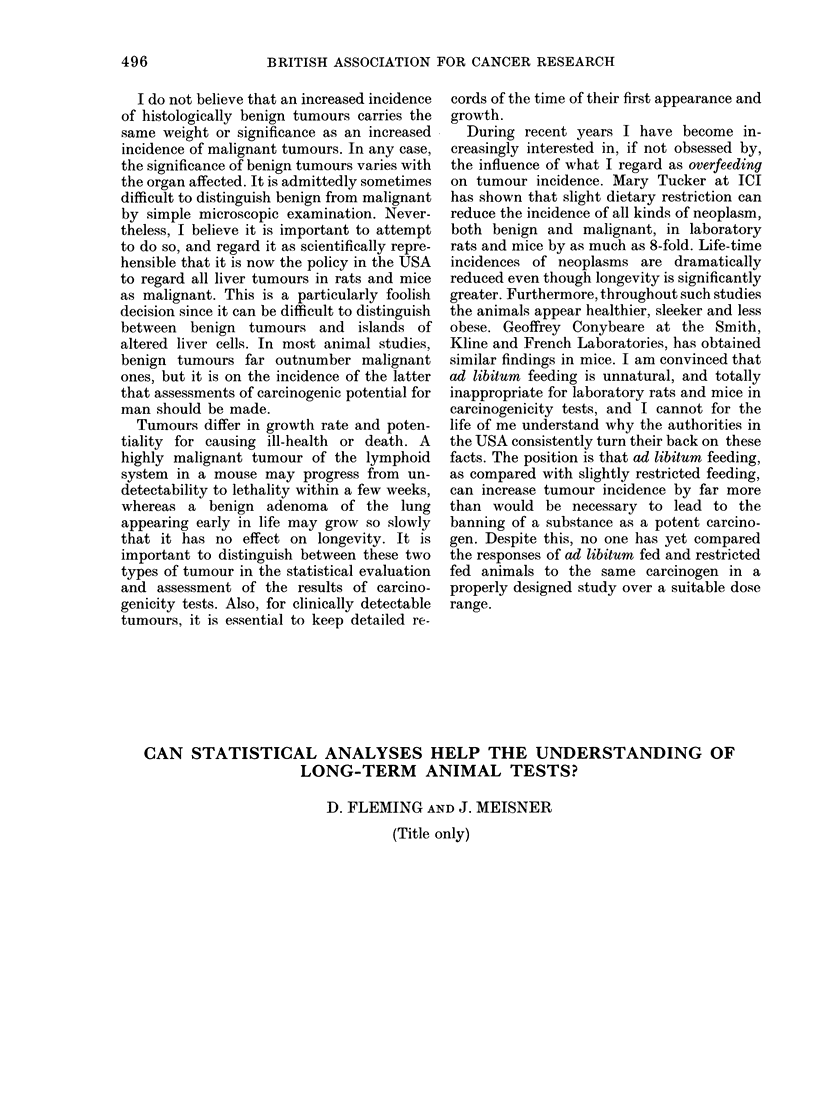# Current methods in testing for carcinogenic activity.

**DOI:** 10.1038/bjc.1980.78

**Published:** 1980-03

**Authors:** F. J. Roe


### CURRENT METHODS IN TESTING FOR CARCINOGENIC ACTIVITY

F. J. C. ROE

*19 Marryat Road, Wimbledon, London*

THIS PAPER concerns the design and interpretation of animal tests for carcinogenicity as one step which may or may not be deemed necessary in the evaluation of chemical substances for carcinogenicity. In designing tests it has to be borne in mind that untreated control animals develop tumours—sometimes in high incidence. Genetic factors, oncogenic viruses and hormonal factors, including those associated with overfeeding, lack of stress, and sex deprivation, are heavily implicated in the aetiology of these background tumours. A cardinal principle of the design of any meaningful test is that apart from the variable under investigation, "like" should be compared with "like", but it may be difficult or impossible to put this principle into practice. For example, inappetance secondary to unpalatability of diet or increasing nonspecific stress associated with increasing dose may pose problems. Alternatively, the administration of a test agent in high dosage may influence hormonal status in a way which alters the risk of tumour development or upsets the balance between an oncogenic virus and its host. For these reasons it is not justifiable to assume, as some workers have, that an increased incidence of any particular type of tumour indicates that the test substance has *induced* the extra tumours by reacting with cellular DNA and thereby transforming normal cells to cancer cells. In other words, an increased incidence of tumours in a long-term animal test does not constitute, by itself, enough information for the purpose of assessing carcinogenic hazard for man. For that purpose information on likely mechanisms is necessary, and it is particularly important to see whether there is continuity or discontinuity in response as one goes down the dose scale. A substance which increases tumour risk in a long-term animal test but gives negative results in bacterial mutagenicity tests may well be acting as a co-carcinogen rather than a carcinogen *in vivo*, although there are several alternative explanations of such a combination of facts.

The objective of carcinogenicity tests should be to obtain the information most useful for assessing likely carcinogenic hazard for man. For this purpose the dose range should extend down to likely human exposure levels, after taking into account absorption, blood levels and other pharmacokinetic data. Carcinogenicity tests confined to dose levels close to maximum tolerated levels may well give misleading positive results, since they provide no information on possible discontinuities in response with decreasing dose.

As far as I am concerned, the more dose levels studied the better, and I usually recommend the inclusion of two separate control groups in any carcinogenicity test which is to be carried out for the purposes of safety evaluation. I believe it is irrational to use strains of rats and mice which are known to be subject to high spontaneous tumour rates, because to do so is to maximize the chances of confusion due to co-carcinogenic effects in such strains. I have been favourably impressed by the suitability of the B6C3/1 hybrid mice widely used for carcinogenicity testing by the National Cancer Institute in its Bioassay Programme. These hybrids appear to be acceptably sensitive to known carcinogens, even though the incidence of "spontaneously-arising" neoplasms in controls is relatively low. The Osborne–Mendel strain of rats used by the National Cancer Institute appears to offer similar advantages.

Ordinarily, exposure to a test substance should begin at weaning and continue through life. Twice-daily surveillance, the killing of sick animals, and a detailed and standard necropsy are the key to meaningful carcinogenicity tests. Tests should normally continue until only, say, 20% of animals survive in the group receiving the highest tolerated dose level, but in this respect the two sexes should be treated separately. I do not like carcinogenicity tests involving transplacental exposure, because of the logistic problems they pose. Also, I know of no substance which has come to be regarded as carcinogenically hazardous solely on the basis of transplacental carcinogenicity.

I do not believe that an increased incidence of histologically benign tumours carries the same weight or significance as an increased incidence of malignant tumours. In any case, the significance of benign tumours varies with the organ affected. It is admittedly sometimes difficult to distinguish benign from malignant by simple microscopic examination. Nevertheless, I believe it is important to attempt to do so, and regard it as scientifically reprehensible that it is now the policy in the USA to regard all liver tumours in rats and mice as malignant. This is a particularly foolish decision since it can be difficult to distinguish between benign tumours and islands of altered liver cells. In most animal studies, benign tumours far outnumber malignant ones, but it is on the incidence of the latter that assessments of carcinogenic potential for man should be made.

Tumours differ in growth rate and potentiality for causing ill-health or death. A highly malignant tumour of the lymphoid system in a mouse may progress from undetectability to lethality within a few weeks, whereas a benign adenoma of the lung appearing early in life may grow so slowly that it has no effect on longevity. It is important to distinguish between these two types of tumour in the statistical evaluation and assessment of the results of carcinogenicity tests. Also, for clinically detectable tumours, it is essential to keep detailed records of the time of their first appearance and growth.

During recent years I have become increasingly interested in, if not obsessed by, the influence of what I regard as *overfeeding* on tumour incidence. Mary Tucker at ICI has shown that slight dietary restriction can reduce the incidence of all kinds of neoplasm, both benign and malignant, in laboratory rats and mice by as much as 8-fold. Life-time incidences of neoplasms are dramatically reduced even though longevity is significantly greater. Furthermore, throughout such studies the animals appear healthier, sleeker and less obese. Geoffrey Conybeare at the Smith, Kline and French Laboratories, has obtained similar findings in mice. I am convinced that *ad libitum* feeding is unnatural, and totally inappropriate for laboratory rats and mice in carcinogenicity tests, and I cannot for the life of me understand why the authorities in the USA consistently turn their back on these facts. The position is that *ad libitum* feeding, as compared with slightly restricted feeding, can increase tumour incidence by far more than would be necessary to lead to the banning of a substance as a potent carcinogen. Despite this, no one has yet compared the responses of *ad libitum* fed and restricted fed animals to the same carcinogen in a properly designed study over a suitable dose range.

### CAN STATISTICAL ANALYSES HELP THE UNDERSTANDING OF LONG-TERM ANIMAL TESTS?

D. FLEMING AND J. MEISNER

(Title only)